# Influence of Heat- and Cold-Stressed Raw Milk on the Stability of UHT Milk

**DOI:** 10.3390/foods14010003

**Published:** 2024-12-24

**Authors:** Nan Li, Zhigang Yang, Zhiyu Yuan, Zizhu Zhen, Xinna Xie, Danqing Zhu, Gang Lu, Feng Zhao, Bo Qu, Bingli Qi, Yujun Jiang, Qianyu Zhao, Chaoxin Man

**Affiliations:** 1 Key Laboratory of Dairy Science, Ministry of Education, Department of Food Science, Northeast Agricultural University, Harbin 150030, China; l2676648333@163.com (N.L.); yu18799@163.com (Z.Y.); zhenzizhu1998@163.com (Z.Z.); 18246812109@163.com (X.X.); zhudanqing2023@163.com (D.Z.); erjinzhi@126.com (F.Z.); qb5172@163.com (B.Q.); yujun_jiang@163.com (Y.J.); 2 Key Laboratory of Infant Formula Food, State Administration for Market Regulation, Harbin 150030, China; 3 Inner Mongolia Mengniu Dairy (Group) Co., Ltd., Hohhot 011500, China; yangzhigang1@mengniu.cn (Z.Y.); lugang@mengniu.cn (G.L.); qibingli@mengniu.cn (B.Q.)

**Keywords:** ultra-high-temperature milk, cold stress, age of gelation, plasmin

## Abstract

This study investigated the variations and alterations in the concentrations of plasmin system components in raw and UHT (ultra-high-temperature) milk under cold stress (WCT ≤ −25 °C), heat stress (THI ≥ 80), and normal (THI < 70 and WCT ≥ −10 °C) circumstances. The findings indicated elevated amounts of plasmin system components in cold-stressed raw milk. While storing UHT milk at 25 °C, the concentrations and activity of plasmin in the milk exhibited an initial increase followed by a decrease, peaking around the 30th day. The maximum plasmin level and activity in cold-stressed milk were 607.86 μg/L and 15.99 U/L, respectively, with the beginning of gelation occurring around day 60. The higher activity of plasmin in cold-stressed milk led to the poorer stability and sensory assessment of the milk. However, heat-stressed milk is not such a problem for UHT milk as cold-stressed milk. The findings indicate shortcomings in the quality of cold-stressed milk and its adverse effects on the stability of UHT milk, underscoring the necessity of preventing cold stress in the herd and refraining from utilizing cold-stressed milk as a raw material for UHT production.

## 1. Introduction

Seasonal variations can induce thermal stress in cattle, adversely affecting their health and milk production. They also influence the quality and stability of UHT (ultra-high-temperature) milk during storage. UHT milk could experience other issues such as gel aging and quality degradation when being stored [1]. Enzymatic and non-enzymatic gels reaction are the two categories of gels identified by the gel aging process. Enzymatic gels are created when heat-resistant proteases hydrolyze casein particles, releasing β-lg-κ-CN complexes into whey that then cross-link to form gels. In contrast, non-enzymatic gels are created when к-CN-depleted micelles gradually agglomerate as a result of gravity while being stored [2,3]. The prolonged gel formation period (usually 12 months) under non-enzymatic conditions significantly surpasses the 6-month shelf life of UHT milk. This study examines the impact of enzymatic processes on milk instability. There are two primary categories of heat-resistant proteases in the enzymatic gelation reaction: exogenous enzymes, exemplified by extracellular proteases released by psychrotrophic bacteria, and endogenous enzymes, which are derived from the bovine organism, primarily consisting of plasmin. The endogenous enzymes primarily originate from the bovine organism itself [4]. This study regulated the quantity and impact of psychrotrophic bacteria spp. The emphasis was on the impact of thermal stress, both hot and cold, on plasmin in milk, as well as on the stability and sensory characteristics of UHT milk.

Plasmin in raw milk exists inside a complex, mutually controlled framework known as the plasmin system. The primary constituents of the plasmin system are plasminogen and plasmin [5,6]. The constituents of the plasmin system in raw milk exist in a state of relative equilibrium; however, the temperature resistance of each constituent varies, resulting in the disruption of this balance during thermal processing. The plasminogen activator inhibitor and plasmin inhibitor exhibit low thermal stability, and both compounds can be inactivated by ultra-high-temperature pre-treatment [7,8,9]. But plasmin, plasminogen, and their activators are more heat-resistant [10], retaining significant activity post-pasteurization, with residual activity detectable even following ultra-high-temperature heat treatment. The leftover plasminogen is perpetually transformed into plasmin by plasminogen activators [11], leading to a sustained elevation in the overall plasmin activity and concentration in milk [12,13]. The hydrolysis of milk proteins occurs during storage, primarily affecting α_s1_-CN, α_s2_-CN, and β-casein in milk [14]; hydrolysis to a specific degree results in the aging of the gel and a decline in quality. A greater residual quantity of plasmin accelerates protein hydrolysis and precipitates gel aging sooner. Moreover, the incidence of thermal stresses predominantly transpires during frigid winters and sweltering summers, with varying seasons significantly influencing the distribution of milk constituents and microorganisms. The protein and fat content of milk components directly dictates the assessment of milk’s sensory attributes.

Consequently, comprehending and regulating the impacts of thermal stress on the concentrations of components within the plasmin system in raw milk, as well as on the quality of UHT milk, are significant [3]. A prior study found that the parameters influencing the constituents of the plasmin system in raw milk largely include the lactation stage, somatic cell count, and mastitis [15]. Nevertheless, there is a paucity of research about seasonal elements that might simultaneously influence the herd in various ways, particularly concerning raw milk quality and the stability and sensory characteristics of UHT milk when cows experience thermal stress from both heat and cold.

Therefore, this study utilized raw milk from farms in northeastern China to examine the impact of cold and heat stress on the plasmin system in raw milk during winter and summer and to assess the levels of its components under varying stress conditions. The raw milk was subsequently processed into UHT milk using UHT processing equipment, followed by packaging and storage. The alterations in plasminogen and plasmin in UHT milk under various stress circumstances were subsequently examined throughout the storage duration, and the impact of plasmin on UHT milk was assessed by evaluating the stability and sensory parameters. This offers specific data assistance for regulating the quality of raw milk at the source, comprehending the influence of plasmin on the stability of UHT milk, and enhancing the shelf life stability of UHT milk.

## 2. Materials and Methods

### 2.1. Materials

Plasmin (lyophilized powder) was obtained from Sigma-Aldrich (St. Louis, MO, USA). MPC (Milk Plate Count Agar) for the quantification of psychrotrophic bacteria and sodium hydroxide (Hope bio-technology, Qingdao, China). The bovine serum plasminogen ELISA kit, bovine serum plasmin ELISA kit (Yuan ju, Shanghai, China), trichloroacetic acid (TCA), and 2-methyl-3-heptanone were acquired from Aladdin (Yuan ye, Shanghai, China). The Triowin PT-20T8 tube sterilizer (Triowin, Shanghai, China). The Thermo X1R cryogenic centrifuge (Thermo scientific, Vacaville, CA, USA). The SpectraMAX I3x Enzyme Labeler (SpectraMAX, Sunnyvale, CA, USA).

### 2.2. Collection of Raw Milk

This study was conducted in northeastern China, distinguished by a chilly continental monsoon climate. Raw milk was sourced from a modern farm housing 9519 Holstein cows, specifically selecting individuals with 2–3 births in mid-lactation. The sampling was controlled for age, the number of births, the lactation stage, medication intervals, nutrition, the health status, and other relevant influencing factors. Thirty samples of raw milk were obtained for each stress condition.

The barn THI (temperature–humidity index) for evaluating summer heat stress in cattle is determined by the formula THI = 0.72 (Td + Tw) + 40.6, where Td signifies the dry bulb temperature and Tw indicates the wet bulb temperature [16,17]. Raw milk with a THI of 80 or above was categorized as heat-stressed milk. The barn WCT (wind chill temperature) index, utilized for evaluating winter cold stress in cattle, is determined by the formula WCT = 13.12 + 0.6215 × T − 11.37 × V^0.16^ + 0.3965 × T × V^0.16^, where the WCT index signifies the wind chill temperature in degrees Celsius (°C), T represents the air temperature in degrees Celsius (°C), and V denotes the wind speed measured at a height of 10 m [18]. Raw milk was classified as cold-stressed milk when the water crystallization temperature (WCT) was less than or equal to −25 °C. Raw milk with a THI < 70 and a WCT ≥ −10 °C was designated as the normal control milk.

### 2.3. Processing of UHT Milk

Employing a tube sterilizer for the ultra-high-temperature sterilization of raw milk, the process involved the filtration of raw milk and homogenization at 25 to 30 MPa, followed by secondary homogenization at 4 to 6 MPa. The preheating section operated at 80 °C with a holding time of about 40 s, the heating section at 137 °C with a holding time of 3 to 4 s, and the cooling section at 25 °C, with a flow rate of 20 L/h. The processed milk was aseptically packed into PET (polyethylene terephthalate) aseptic bottles and stored at consistent temperatures of 4 °C, 25 °C, and 37 °C, sheltered from light.

### 2.4. Determination of Milk Composition

The composition of the milk, the colony count, and the somatic cell count of raw milk were assessed using the CombiFoss FT + analyzer (Foss Allé, Hillerød, Denmark) [19]; the study of the milk composition encompassed the protein, fat, lactose, total solids, non-fat milk solids, freezing point, somatic cell count, and total colony count. Fifty milliliters of cow’s milk samples were collected in a sample bottle and examined three times, and the average value was recorded.

### 2.5. Determination of Plasminogen and Plasmin

Plasminogen and plasmin were quantified in milk samples utilizing ELISA, It is called a solid-phase enzyme immunoassay because it employs an enzyme-linked antigen or antibody as a marker for the detection of the analyte of interest. In the ELISA, an antigen must be immobilized on a solid surface and then complexed with an antibody that is linked to an enzyme [20]. We obtained 10 mL of milk samples, extracted the fat, took 5 mL of skimmed milk, added 150 μL of 17.2% potassium ferricyanide, shook it, then added 150 μL of 53.5% zinc sulfate, vortexed it, centrifuged it at 3500× *g* for 10 min, and then collected the supernatant and combined it with an equal volume of a PBS buffer, ensuring thorough mixing for measurement. We transferred 50 μL of the solution to be analyzed into the antibody-coated microtiter plate for warm incubation. Following washing and color development, the absorbance (optical density value) was assessed at 450 nm, and the concentration and activity of bovine plasminogen or plasmin in the samples were derived using calculations based on the standard curve. The blank control was substituted with PBS, and each sample was replicated three times, with the mean value calculated.

### 2.6. Determination of Titratable Acidity

The titration method employed phenolphthalein as an indicator, utilizing a 0.1000 M sodium hydroxide standard solution to titrate a 100 g sample until the endpoint was reached. Subsequent calculations were performed to ascertain the sample’s acidity based on the volume of the sodium hydroxide solution consumed [21].

### 2.7. The Determination of the Total Number of Psychrotrophic Bacteria

We utilized MPC media. Five distinct dilutions of raw milk were prepared, with 1 mL of each dilution applied to a sterile Petri dish. The requisite volume of the medium was then introduced into each dish, which was subsequently incubated aerobically at 6.5 °C for 10 days for colony enumeration.

### 2.8. Determination of Centrifugal Sedimentation Rate

The centrifugal sedimentation rate (CSR) of the samples was established following the methodology of Jensen S [22]. Following the homogenization of the mixed protein solution, 10 g of the samples (m_0_) was precisely measured and centrifuged at 4000 r/min for 15 min, and the weight of the sediment (m_1_) at the base of the centrifugation tubes was recorded. The centrifugal sedimentation rate was determined by conducting three repetitions for each sample.

CSR (%) = (m_1_/m_0_) × 100%

### 2.9. Determination of Protein Hydrolysis (DH, %)

The DH of the samples was ascertained using Cui’s methodology [23]: The 400 μL samples were combined with 3 mL of O-phthalaldehyde (OPA) reagent at an ambient temperature (22–28 °C), followed by precise incubation for 2 min. The absorbance of the combined solution was quantified using SpectraMAX I3x Enzyme Labeler at 340 nm.

### 2.10. Stability Measurement

A vertical analysis of UHT milk in the cuvette was conducted to assess its stability with the emulsion stability analyzer TURBISCAN Lab [24]. The borosilicate glass cuvette had an inner diameter of 20 mm and a height of 40 mm. The measurements were conducted at 25 °C, with a 15 min gap between transmitted and backscattered light assessments for a duration of 24 h. The horizontal axis denoted the height of the cuvette, while the vertical axis also indicated the height of the cuvette. The horizontal axis denoted the elevation of the injection cell, while the vertical axis indicated the intensity of the backscattered light. The first scan was depicted in blue, while the final scan was represented in red.

### 2.11. Determination and Analysis of Color, Flavor, Odors, and Volatile Substances

#### 2.11.1. Color Detection

The CIE parameters L*, a*, and b* were assessed in milk samples utilizing a ColorFlex EZ colorimeter. L* denotes the brightness of the sample, with positive values indicating brightness and negative values indicating darkness. a* reflects the sample’s hue, with positive values reflecting redness and negative values indicating greenness. b* signifies the yellowish-blue spectrum, where an increase in the value corresponds to a transition towards yellow or brown hues. ΔE was computed using the acquired L*, a*, and b* variables.

The alteration in the color of liquid milk during storage was represented as the color difference ΔE, which is computed using the following formula:

$$\Delta E=\sqrt{{\left({\Delta L}^{*}\right)}^{2}+{\left({\Delta a}^{*}\right)}^{2}{+({\Delta b}^{*})}^{2}}$$

where ΔL*, Δa*, and Δb* represent the differences between the measured values of L*, a*, and b* and the original measured values during the storage of milk, respectively.

#### 2.11.2. Electronic Tongue Determination

This approach cites Qin [1]: 35 mL of liquid milk was introduced into the sample cup of the electronic tongue (INSENT, Kyushu, Japan) and sealed at an ambient temperature for 20 min. The testing method commenced following the self-testing and calibration of the device, with each sample measured four times.

#### 2.11.3. Electronic Nose (E-Nose) Measurement

This approach cites Liu et al. [25]. An electronic nose (PEN-3, Schwerin, Mecklenburg, Germany) was utilized: 30 mL of the sample was placed in a beaker, 15 mL was aspirated into a headspace injection bottle, the sensor flow rate was 230 mL/min, the carrier gas flow rate was 200 mL/min, the cleaning duration was 60 s, the sampling interval of the electronic nose was 1 s, and the detection duration was 60 s. The sample was collected concurrently, although it was not introduced into the headspace injection vial.

#### 2.11.4. Determination of Volatile Substances

This approach cites Clarke [26]. Five grams of the emulsion was measured into a headspace vial, with 2-methyl-3-heptanone employed as the internal standard. The samples were extracted at 60 °C for 45 min. Each sample underwent pre-extraction at 60 °C for 10 min, accompanied by pulsed stirring at 500 rpm for 5 s. Sampling was conducted using a manual sampler.

Volatiles were extracted utilizing a Divinylbenzene/carbon molecular sieve/polydimethylsiloxane SPME fiber. Post-extraction, the column was injected at 250 °C for 5 min in split-flow mode with a ratio of 10:1. The column oven was held at 65 °C for 10 min and thereafter increased to 240 °C at a rate of 10 °C per minute and sustained for 4 min. Helium served as the carrier gas at a consistent flow rate of 1.0 mL/min.

The mass spectrometry parameters included the following: a medium-polarity DB-MS column (60 m × 0.32 mm × 1.80 µm) electron bombardment ionization source with the ionization voltage set at 70 eV, the transmission line temperature maintained at 250 °C, the ion source temperature at 230 °C, the four-stage rod temperature at 150 °C, a solvent delay of 3 min, mass scanning conducted in full-scan mode, and a mass scanning range of *m*/*z* 30–500.

### 2.12. Statistical Analysis

Data were analyzed using a one-way ANOVA with SPSS Statistics 20.0 software (SPSS Inc., Chicago, IL, USA). The results were presented as the mean ± standard deviation. Statistical significance was established at *p* < 0.05. A multivariate statistical data analysis was developed for the analysis and interpretation of metabolite data. The generated data underwent standardization and normalization through logarithmic transformation and Pareto scaling. Principal component analysis (PCA) was employed for unsupervised statistical analysis to classify and distinguish the groups.

## 3. Results and Discussion

### 3.1. Physicochemical and Biological Indicators of Raw Milk

**Figure 1:**
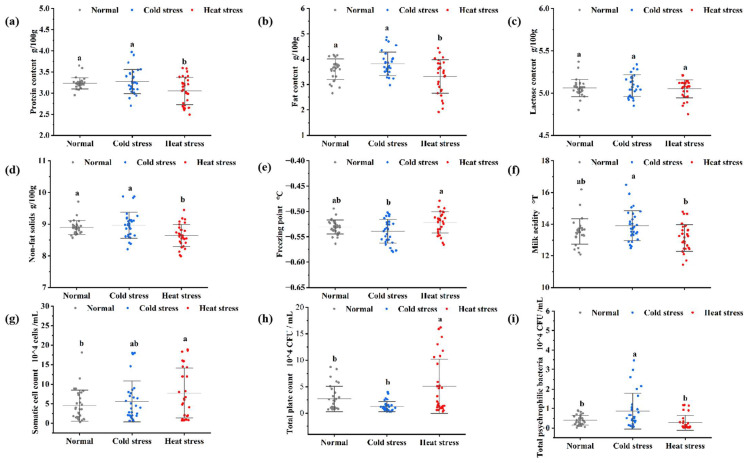
Physicochemical and biological indicators of raw milk. Note: (**a**) protein content, (**b**) fat content, (**c**) lactose content, (**d**) non-fat milk solids content, (**e**) freezing point, (**f**) milk acidity, (**g**) somatic cell count, (**h**) total colony count, and (**i**) total psychrotrophic bacteria count. Distinct lowercase letters signify statistically significant variations among the data (*p* < 0.05). Various colors in the diagrams on this page distinguish milk under different stress conditions; the gray data points signify normal milk, which is the unaltered milk produced when the cow is not subjected to thermal stress. The blue data points denote raw milk produced by cows experiencing cold stress, while the red data points denote raw milk produced by cows experiencing heat stress. There were 30 samples of each variety of raw milk, resulting in a total of 30 data points.

The physicochemical and biological indicators of raw milk influenced by seasonal heat and cold stress [17,18] are shown in Figure 1a–d. The protein, fat, lactose, and non-fat solid contents of cold-stressed milk were greater, accordingly, than those of hot-stressed milk, possibly attributable to the cows’ food intake and feed composition. This is probably associated with the quantity of food and the composition of the cows’ feed [27]. Furthermore, the reduced feed intake and heightened water consumption of cows experiencing heat stress contributed to the diminished milk composition [28].

The freezing point and acidity served as physicochemical markers of milk, tested with the CombiFoss FT + analyzer (Foss Allé, Hillerød, Denmark). Figure 1e,f demonstrate that cold-stressed milk had a reduced mean freezing point, differing by approximately 0.0175 °C from the mean freezing point of hot-stressed milk. The concentration of solids in milk influences osmolality, a phenomenon attributable to the higher milk solids content in cold-stressed milk. Previous research confirmed a correlation between freezing point and seasonal variations, as well as the quantity of lactose and non-fat milk solids in milk, the freezing point range is also similar [29]. There were higher mean values of acidity in cold-stressed milks [30], about 0.78 °T higher than the average value of heat stress lactic acidity. Milk acidity is influenced by the natural and fermentation acidity; in this study, preservation conditions were regulated and the fermentation acidity was minimal, so only the natural acidity was taken into account. The natural acidic constituents in raw milk include casein, albumin, phosphate, citrate, and carbonate, with casein and soluble phosphorus comprising about 80% of the titratable acidity of milk [31]. Thus, the higher acidity of cold-stressed milk can be related to the higher protein concentration.

Biological indicators signify the sanitation and health of raw milk. Figure 1g shows that higher somatic cell counts in milk subjected to heat stress, could be ascribed to the diminished bodily condition and immunity of cows experiencing such conditions [32]. Figure 1h,i show that heat-stressed milk exhibited colony counts approximately 88% and 260% greater than those of normal and cold-stressed milks, respectively, but the quantity of psychrotrophic bacteria was quite minimal. Cold-stressed milk contained low levels of colony counts but high numbers of psychrotrophic bacteria, approximately 114% and 214% greater than the average values of normal and heat-stressed milk, respectively. The elevated colony counts can be ascribed to the rapid proliferation of microorganisms within the enclosure and on the bedding, facilitated by the humid and warm conditions of summer [33]. This significantly elevates the likelihood that bacteria will get into contact with the cow’s udder and contaminate the raw milk [34]. The increased prevalence of psychrotrophic bacteria can be ascribed to the lower winter temperatures, which are more conducive to their development and reproduction [35].

### 3.2. Raw Milk Plasmin System Components

**Figure 2:**
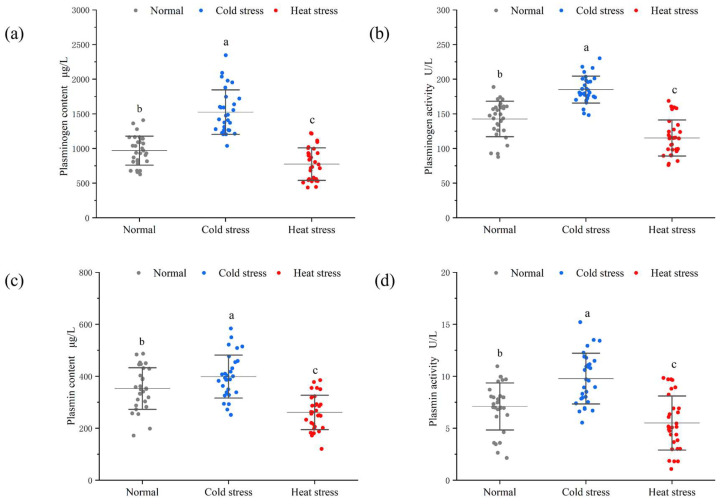
Plasmin system components in raw milk under different stress conditions. (**a**) Plasminogen content, (**b**) plasminogen activity, (**c**) plasmin content, and (**d**) plasmin activity. Every data point signifies a sample of unprocessed milk. Distinct letters (a–c) within the identical class signify significant variations (*p* < 0.05).

Mastitis in cows compromises the blood–milk barrier of the mammary gland, resulting in heightened permeability, allowing the components of the plasmin system in the blood to infiltrate the milk via the junctions of the mammary cells [36,37]. (Figure 2) The elevated levels and activity of plasminogen and plasmin in cold-stressed milk can be ascribed to the fact that cows under cold stress allocate most of their bodily energy to combating the cold, resulting in diminished immunity. Consequently, these cows exhibit insufficient resistance to microbial environments, particularly against *Escherichia coli*, environmental streptococci, and other pathogens, making them susceptible to clinical mastitis [38,39]; immune cells stimulated by mastitis can release substantial quantities of the plasminogen activator [40]. Furthermore, increased milk production during winter can lead to prolonged mechanical milking, which may harm the udder and permit blood to contaminate the milk, consequently elevating the concentration of the plasmin system in the milk [41,42]. Elevated lactation levels can induce physiological stress in cows, adversely impacting the immune system and increasing the susceptibility to a teat injury, hence raising the prevalence of mastitis [43,44].

### 3.3. Changes in the Components of the Plasmin System During the Storage of UHT Milk

**Figure 3:**
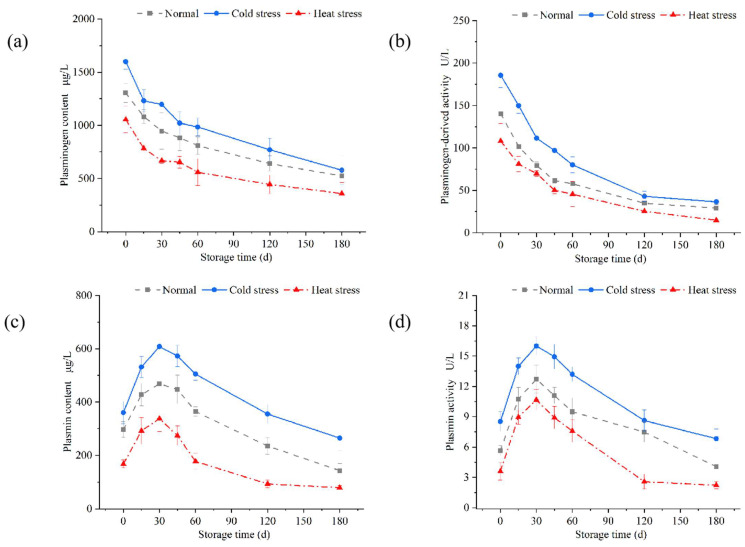
Changes in the components of the plasmin system during the storage of UHT milk at 25 °C. (**a**) Plasminogen content, (**b**) plasminogen activity, (**c**) plasmin content, and (**d**) plasmin activity. Data are presented as mean ± SD of triplicates.

During the storage of UHT milk at 25 °C, the residual plasminogen can be transformed into plasmin, a process characterized by its dynamic nature. UHT processing does not fully inactivate the constituents of the plasmin system [9,45]. The concentration and activity of plasmin were elevated in milk subjected to cold stress (Figure 3). The plasmin concentration and activity in cold-stressed milk were elevated, peaking at approximately 30 days of storage, with values of 607 μg/L and 15.9 U/L (Figure 3c,d), respectively. The increased protease concentration and activity in UHT milk accelerated the protein breakdown. Consequently, cold-stressed UHT milk was at the higher risk of gel aging due to the hydrolysis of plasmin over the storage duration. Plasminogen persistently diminished due to activated conversion, while the plasmin content and activity surged swiftly to their peak during the pre-storage phase and subsequently declined progressively in the storage period owing to the autolytic effects of plasmin [46].

### 3.4. The Hydrolysis and Precipitation of Milk Proteins During the Storage of UHT Milk

**Figure 4:**
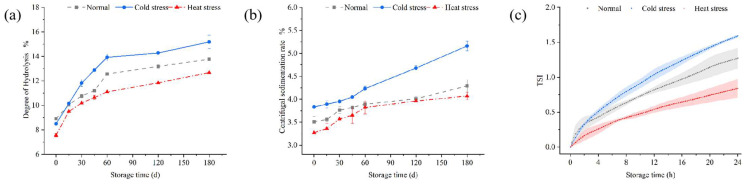
Hydrolysis of plasmin and its impact on the stability of UHT milk. (**a**) Changes in hydrolysis degree and (**b**) centrifugal sedimentation rate of UHT milk during storage under different stress conditions. (**c**) Kinetic instability index (TSI) of UHT milk under different stress conditions. The value of the detection error is indicated by the range of extension around the connecting line. Data are presented as mean ± SD of triplicates.

Protein hydrolysis and the centrifugal sedimentation rate were employed to evaluate the degree of milk protein hydrolysis and solid precipitation. Figure 4a illustrates the fluctuation of milk protein hydrolysis over the storage duration of UHT milk at 25 °C, with cold-stressed milk exhibiting the greatest hydrolysis rate of 15.18%. This could have been due to the elevated levels of plasmin and proteins in UHT cold-stressed milk, resulting in a more rapid increase in the degree of hydrolysis during the storage cycle. During the storage period, plasmin-mediated protein hydrolysis resulted in the degradation of some casein components in the milk, mostly yielding various breakdown products, notably β-casein and αs1-casein, which underwent extensive hydrolysis [9]. The ongoing hydrolysis resulted in a reduction in the protein content and an elevation in the degree of hydrolysis [2,47]. Figure 4b illustrates the variations in the centrifugal sedimentation rate over time. The centrifugal sedimentation rate of UHT milk under varying stress conditions progressively increased with a prolonged storage time, with cold-stressed milk exhibiting a more rapid and pronounced increase during the latter storage phase, ultimately reaching 4.68% at 120 days of storage. This phenomenon was due to the hydrolysis of cold-stressed milk by potent plasmin, resulting in an increased degree of hydrolysis, leading to the progressive aggregation and precipitation of milk solids. This can be ascribed to the escalating extent of hydrolysis and the progressive agglomeration and precipitation of milk solids [2]. With the extension of hydrolysis time, the degree of hydrolysis escalated, resulting in the further decomposition or aggregation of milk proteins. The particle size of the milk augmented with the elevation in the degree of hydrolysis. This influences the consumer’s preference for milk to some degree.

The TSIs (Turbine Supervisory Instruments) index is derived by integrating light intensity measurements with the kinetic formula. A greater value signifies the increased instability of the system [48]. Figure 4c illustrates the stability of milk samples subjected to various stress conditions during mid-term storage at 25 °C. The TSI value for cold-stressed milk exceeded that of both normal and heat-stressed milk, with the final scan’s TSI index reaching 1.59, in contrast to 1.27 for normal milk and 0.84 for heat-stressed milk. This discrepancy may be ascribed to variations in the milk composition and plasmin hydrolysis, which contributes to the instability of the milk system.

### 3.5. Changes in Color, Flavor, Odors, and Volatile Substances of UHT Milk During Storage

The storage temperature is an important factor affecting the color, flavor, odors, and volatile substances of UHT milk during the same storage time. Therefore, three different temperatures of 4 °C, 25 °C, and 37 °C were set for UHT milk storage, and the color difference, taste, odor, and volatile substances of UHT milk with three different conditions were measured after 6 months of storage.

#### 3.5.1. Color Difference Analysis

**Figure 5:**
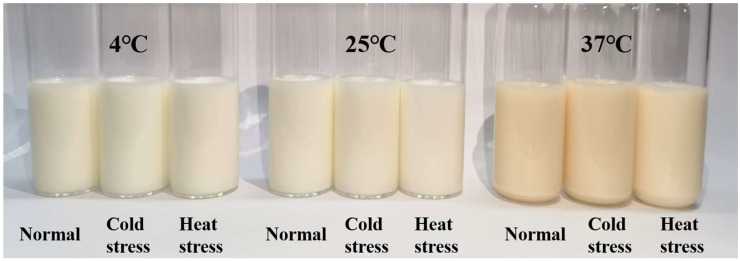
Comparison of color difference in UHT milk under different temperature storage conditions.

The milk’s color variation was assessed using a colorimeter, which computed ΔE based on the recorded L*, a*, and b* values. The ΔE value quantified the color variation in UHT milk following storage under various temperature conditions. Fresh cow’s milk is a white opaque emulsion [49], with its hue primarily determined by the fat content [50]. Table 1 suggests that the color difference values of cold-stressed milk were significantly greater (*p* < 0.05) across all three storage temperature conditions, with a ΔE of 14.54 at 37 °C. The higher milk fat content in cold-stressed milk, along with the higher plasmin activity that hydrolyzed milk fat globule membrane proteins, resulted in lipoglobulin aggregation, thereby intensifying the impact on the milk color [51]. Moreover, elevated storage temperatures prolonged the Maillard reaction, resulting in an increased ΔE value of the milk [52]. A more intuitive display of color variations, Figure 5 illustrates the actual outcomes of the three distinct strained milks during storage at 37 °C.

#### 3.5.2. Flavor

**Figure 6:**
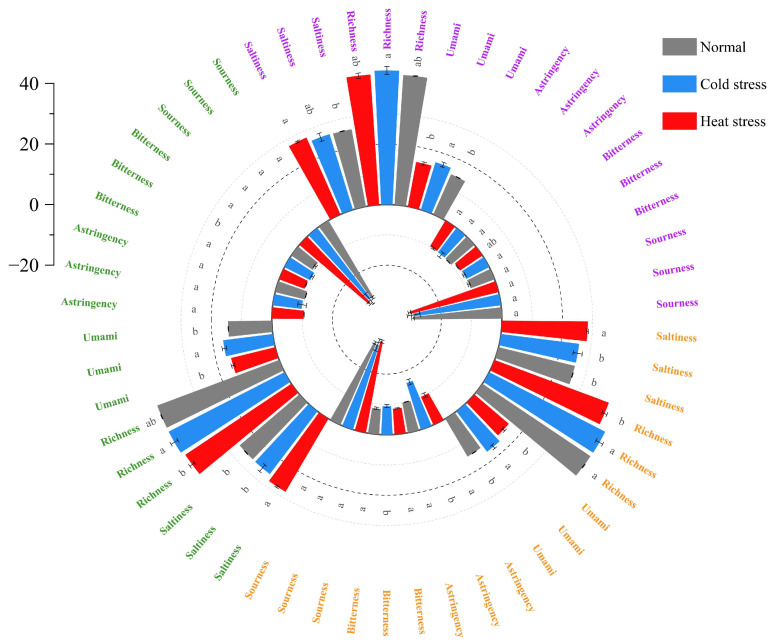
Flavor profiles of UHT milk subjected to various stress conditions following storage at diverse temperatures. Various text colors denote distinct storage temperatures. The purple letters denote UHT milk maintained at 4 °C, the green letters denote UHT milk maintained at 25 °C, and the orange letters denote UHT milk maintained at 37 °C. Data are expressed as mean ± standard deviation of triplicates. Distinct letters (a, b) within the identical class signify significant variations (*p* < 0.05).

The characterization of flavor alterations in milk was performed by the electronic tongue, where varying potential values were associated with distinct taste profiles. As shown in Figure 6, under the storage condition of 4 °C, the relative umami and richness of cold-stressed milk were more significant (*p* < 0.05). Under the storage condition of 25 °C, the relative bitterness, umami, and richness of cold-stressed milk were more significant (*p* < 0.05). Under the storage condition of 37 °C, the relative bitterness, astringency, umami, and richness of cold-stressed milk were more significant (*p* < 0.05). For hot-stressed milk, it was the saltiness that was more significant at all three storage temperatures. Bitterness in UHT milk is associated with protein degradation [2,53]. The peptides formed by the hydrolysis of proteins by plasmin contain many bitter peptides, which are mainly derived from α_S1_ casein and α_S2_ casein. In addition, acetic acid and furfural generated by the Maillard reaction are also associated with bitterness [54], and the intermediates of lipid oxidation are also involved in the Maillard reaction and promote non-enzymatic browning, producing more furfural [55]. The bitterness and astringency of cold-stressed milk in the figure can be attributed to the high protein, fat, and lactose content of cold-stressed milk, as well as more intense hydrolysis by plasmin [56].

#### 3.5.3. Odors

**Figure 7:**
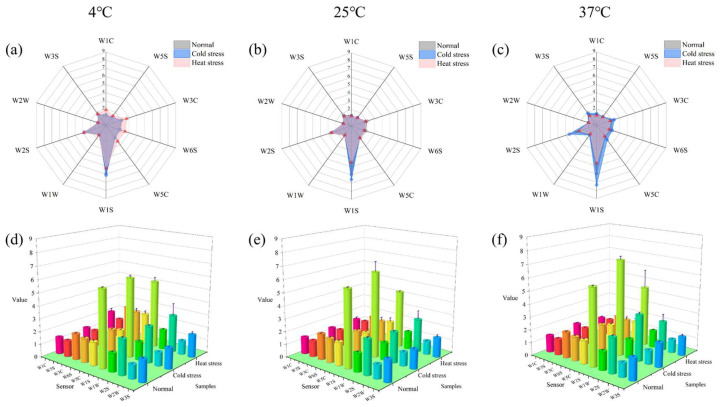
Radar charts and 3D bar charts illustrate the classes of odorants in milk, specifically the odorants of UHT milk subjected to various stress situations in different storage environments. (**a**) Odor distribution radar plots of three conditions of milk under storage condition at 4 °C. (**b**) Odor distribution radar plots of three conditions of milk under storage condition at 25 °C. (**c**) Odor distribution radar plots of three conditions of milk under storage condition at 37 °C. (**d**) Histogram of the odor distribution of the three conditions of milk under storage condition at 4 °C. (**e**) Histogram of the odor distribution of the three conditions of milk under storage condition at 25 °C. (**f**) Histogram of the odor distribution of the three conditions of milk under storage condition at 37 °C.

An electronic nose was used to detect the odor of milk and all 10 sensors of the electronic nose responded, and the responsive substances are shown in Table 2. As shown in Figure 7, for milk under three different stress conditions at the end of storage at different storage temperatures, the ammonia and aromatic molecule, hydride, olefin, aromatic, polar molecule, alkane, alcohol, and partially aromatic compound sensors detected significantly higher values (*p* < 0.05), and the higher the storage temperature, the greater the quantity of alkanes, alcohols, and partially aromatic compound flavor substances generated. The significantly higher (*p* < 0.05) values in the cold-stressed milk than in the other two groups could be attributed to the higher content of fat and protein in the cold-stressed milk and the release of more alkane flavor substances. In the comparison of ammonia and aromatic, hydride, olefinic, aromatic, and polar molecules, it was found that the content value of heat-stressed milk was significantly higher (*p* < 0.05) than the other two groups at 4 °C storage condition, but the content value of cold-stressed milk was significantly higher (*p* < 0.05) than the other two groups at the 37 °C storage condition. This is consistent with the GC-MS results: the main flavor substances in heat-treated milk were aldehydes, fatty acids, alcohols, ketones, and sulfur compounds.

#### 3.5.4. Volatile Substance

The volatile compounds in milk were identified using GC-MS, and the results are shown in Table 3, Table 4 and Table 5. A total of 14 volatile compounds were detected in all samples. Among them, six volatile compounds were identified in normal milk, six in cold-stressed milk, and five in heat-stressed milk at 4 °C, all of which were ketones and alkanes. When stored at 25 °C, five volatile compounds were identified in normal milk, nine in cold-stressed milk, and five in heat-stressed milk, all of which were aliphatic ketones and alkanes. At 37 °C, seven volatile compounds were identified in normal milk, eight in cold-stressed milk, and seven in heat-stressed milk, including aldehydes, ketones, and alkanes.

Ketones are flavor compounds found in bovine dairy products [57]. The flavors of 2-heptanone and 2-nonanone are common. For example, 2-heptanone and 2-nonanone present creamy and sweet flavors [58]. From Table 5, it can be seen that the higher the storage temperature, the more volatile substances there were, which may have been closely related to fat oxidation. Alkanes are compounds formed in milk when it is heated [59]. Different storage temperatures result in the different volatilization of alkanes in cow’s milk, and the higher the storage temperature, the more pronounced the volatilization. At the same time, the hydrolysis of fat globule membrane proteins by plasmin during the storage period caused the imbalance and aggregation of milk lipids [51], which further promoted the oxidation of milk fat as well as the alteration of flavor substances.

Aldehydes are the principal secondary products of the autoxidation of unsaturated fatty acids, while hydroperoxides serve as their primary products, which can subsequently decompose into hydrocarbons, alcohols, and carbon-based compounds [60]. Aldehydes, intermediates, and unstable chemicals are typically converted to alcohols and are present in low amounts in the volatile fraction of most skimmed milk samples, yielding results consistent with prior studies. Aldehydes are not commonly present in milk, and their low concentrations suggest optimum processing conditions. Aldehydes in low quantities exhibit a scent reminiscent of green grass-like herbs and enhance the fresh taste of milk; nevertheless, at elevated concentrations, they can produce undesirable odors [61].

Alkanes are the predominant chemicals generated during the preheating process in milk [59]. Research indicates that heptane, octane, decane, and undecane exhibit the highest amounts in yogurt derived from cow, buffalo, ewe, and sheep milk. The concentration of alkane compounds is almost irrelevant to the flavor due to the high thresholds [60]. Alkanes could originate from forages.

Table 3, Table 4 and Table 5 illustrate that cold-stressed milk has a greater variety of volatile compounds, including aldehydes, ketones, alkanes, and other volatile substances, at the same storage temperature, with the relative concentration of most volatile compounds being elevated. This can be ascribed to the impact of cold stress on the eating and digestion of dairy cows [62].

## 4. Conclusions

This paper provides an in-depth discussion on the alteration of some milk components and plasmin system fractions in the raw milk of Holstein cows during hot and cold stress and investigates the changes in cold- and heat-stressed UHT milk subjected to hydrolysis by plasmin during the storage period. The experimental results included the higher contents of milk components such as protein and fat as well as plasmin system components in cows during hot and cold stress. After UHT treatment, cold-stressed milk in storage was subjected to hydrolysis by more active plasmin, resulting in a higher protein hydrolysis, centrifugal sedimentation rate, and milk instability index. Moreover, the color change of cold-stressed milk was more obvious after storage, and the bitterness, astringency, umami, and richness were stronger. This study improves the understanding of raw milk quality, as well as the storage stability of UHT milk during hot and cold stress in dairy cows, and provides some reference for improving the quality control of raw milk and the storage stability of UHT milk.

## Figures and Tables

**Table 1:** Color difference in milk after storage.

Color Parameter Values After Storage				
Parametric	Sample	4 °C	25 °C	37 °C
ΔE	Normal	5.39 ^a^	7.53 ^a^	10.01 ^b^
	Cold stress	6.29 ^a^	8.75 ^a^	14.54 ^a^
	Heat stress	4.55 ^a^	6.10 ^a^	9.69 ^b^

Note: Different letters in the same row indicate significant differences in the samples (*p* < 0.05). ΔE is based on standard colorimetric whiteboards.

**Table 2:** Name of electronic nose sensor and its corresponding components.

Sensor Name	Performance Description
W1C	Aromatic compounds
W5S	Reacts to nitrogen oxides
W3C	Sensitive to ammonia, aromatic ingredients
W6S	Mainly sensitive to hydrogen
W5C	Sensitive to alkane and aromatic compounds, less so to polar compounds
W1S	Mainly sensitive to hydrocarbons
W1W	Mainly sensitive to sulfide compounds
W2S	Sensitive to alcohol, reacts to carbon group
W2W	Sensitive to aromatic compounds and sulfur organic compounds
W3S	Reacts to alkanes and selective methane

**Table 3:** Relative percentage of volatiles under 4 °C storage condition.

	Categorization		Normal				Cold Stress				Heat Stress			
Form	Name of Substance	CAS	RT	Peak Area	Relative Percentage	Relative Content	RT	Peak Area	Relative Percentage	Relative Content	RT	Peak Area	Relative Percentage	Relative Content
Ketones	3,4-Dimethyl-2-heptanone	196106-96-4	5.94	0.26	0.35	10.43	-	-	-	-	-	-	-	-
	2-Heptanone	110-43-0	4.16	0.97	1.29	38.57	4.49	5.82	39.89	11.07	-	-	-	-
	2-Nonanone	821-55-6	13.45	0.42	0.56	16.83	13.56	3.78	25.95	7.21	13.77	0.25	18.31	3.74
	2-Undecanone	112-12-9	18.04	0.14	0.18	5.48	18.06	1.67	11.48	3.19	16.21	0.02	1.77	0.36
Alkanes	Tetradecane	90622-46-1	19.71	0.03	0.04	1.20	19.73	0.17	1.14	0.32	-	-	-	-
	Tridecanone	593-08-8	-	-	-	-	21.04	1.05	7.23	2.01	-	-	-	-
	2-Methy- ldodecane	1560-97-0	23.53	0.08	0.11	3.30	-	-	-	-	-	-	-	-
	Pentadecane	629-62-9	-	-	-	-	-	-	-	-	15.78	0.05	3.79	0.77
	Hexadecane	544-76-3	-	-	-	-	-	-	-	-	21.10	0.15	11.07	2.26
	n-Hexade -canoic acid	67701-02-4	-	-	-	-	-	-	-	-	26.31	0.07	5.15	1.05
	Heneicosane	629-94-7	-	-	-	-	20.95	0.19	1.31	0.36	-	-	-	-

**Table 4:** Relative percentage of volatiles under 25 °C storage condition.

	Categorization		Normal				Cold Stress				Heat Stress			
Form	Name of Substance	CAS	RT	Peak Area	Relative Percentage	Relative Content	RT	Peak Area	Relative Percentage	Relative Content	RT	Peak Area	Relative Percentage	Relative Content
Aldehydes	Heptaldehyde	111-71-7	-	-	-	-	4.81	0.63	3.24	0.78	-	-	-	-
	2-Heptanone	110-43-0	-	-	-	-	4.44	10.80	55.69	13.42	4.29	4.53	67.37	2.92
	2-Nonanone	821-55-6	13.77	0.25	18.31	3.74	13.54	2.22	11.43	2.75	13.51	0.41	6.07	0.26
	2-Undecanone	112-12-9	16.21	0.02	1.77	0.36	18.05	1.62	8.34	2.01	18.04	0.62	9.23	0.40
Alkanes	Tetradecane	90622-46-1	-	-	-	-	16.33	0.66	3.40	0.82	-	-	-	-
	Tridecanone	593-08-8					21.03	0.61	3.13	0.75				
	2-Methy- ldodecane	1560-97-0	-	-	-	-	23.53	0.54	2.80	0.68	-	-	-	-
	Pentadecane	629-62-9	15.78	0.05	3.79	0.77	-	-	-	-	19.72	0.17	2.48	0.11
	Heptadecane	629-78-7	-	-	-	-	-	-	-	-	23.53	0.25	3.71	0.16
	Hexadecane	544-76-3	21.10	0.15	11.07	2.26	-	-	-	-	-	-	-	-
	n-Hexade- canoic acid	67701-02-4	26.31	0.07	5.15	1.05	26.30	0.23	1.18	0.29	-	-	-	-
	Heneicosane	629-94-7	-	-	-	-	22.35	0.16	0.81	0.19	-	-	-	-

**Table 5:** Relative percentage of volatiles under 37 °C storage condition.

	Categorization		Normal				Cold Stress				Heat Stress			
Form	Name of Substance	CAS	RT	Peak Area	Relative Percentage	Relative Content	RT	Peak Area	Relative Percentage	Relative Content	RT	Peak Area	Relative Percentage	Relative Content
Aldehydes	Nonanal	124-19-6	-	-	-	-	13.16	0.09	1.21	1.24	-	-	-	-
Ketones	3,4-Dimethyl-2-heptanone	196106-96-4	3.10	0.36	4.80	4.93	-	-	-	-	-	-	-	-
	2-Heptanone	110-43-0	-	-	-	-	5.17	13.35	42.96	55.46	4.47	1.34	28.67	3.92
	2-Nonanone	821-55-6	12.58	1.38	18.57	19.11	13.80	2.69	8.66	11.19	13.55	0.18	3.76	0.51
	2-Undecanone	112-12-9	17.90	0.55	7.39	7.60	18.09	11.98	38.56	49.77	18.06	0.21	4.57	0.62
Alkanes	Tetradecane	90622-46-1	19.66	0.26	3.50	3.60	-	-	-	-	19.73	0.08	1.64	0.22
	Tridecanone	593-08-8	-	-	-	-	20.99	0.21	2.88	2.96	-	-	-	-
	Pentadecane	629-62-9	-	-	-	-	21.06	0.34	4.55	4.68	-	-	-	-
	Heptadecane	629-78-7	22.33	0.19	2.56	2.64	-	-	-	-	-	-	-	-
	Hexadecane	544-76-3	23.51	0.23	3.04	3.13	-	-	-	-	21.10	0.48	10.31	1.41
	n-Hexade -canoic acid	67701-02-4	26.33	0.68	2.19	2.83	26.29	2.57	34.58	35.58	26.30	0.21	4.50	0.62
	Heneicosane	629-94-7	-	-	-	-	22.36	0.43	1.39	1.80	22.36	0.08	1.73	0.24

## Data Availability

The data presented in this study are available on request from the corresponding author due to the original contributions presented in the study are included in the article.
